# Fair Balance and Adequate Provision in Direct-to-Consumer Prescription Drug Online Banner Advertisements: A Content Analysis

**DOI:** 10.2196/jmir.5182

**Published:** 2016-02-18

**Authors:** Crystal Adams

**Affiliations:** ^1^ University of Miami Coral Gables, FL United States

**Keywords:** direct-to-consumer advertising, prescription drugs, Internet, pharmaceutical policy, United States Food and Drug Administration

## Abstract

**Background:**

The current direct-to-consumer advertising (DTCA) guidelines were developed with print, television, and radio media in mind, and there are no specific guidelines for online banner advertisements.

**Objective:**

This study evaluates how well Internet banner ads comply with existing Food and Drug Administration (FDA) guidelines for DTCA in other media.

**Methods:**

A content analysis was performed of 68 banner advertisements. A coding sheet was developed based on (1) FDA guidance documents for consumer-directed prescription drug advertisements and (2) previous DTCA content analyses. Specifically, the presence of a brief summary detailing the drug’s risks and side effects or of a “major statement” identifying the drug’s major risks, and the number and type of provisions made available to consumers for comprehensive information about the drug were coded. In addition, the criterion of “fair balance,” the FDA’s requirement that prescription drug ads balance information relating to the drug’s risks with information relating to its benefits, was measured by numbering the benefit and risk facts identified in the ads and by examining the presentation of risk and benefit information.

**Results:**

Every ad in the sample included a brief summary of risk information and at least one form of adequate provision as required by the FDA for broadcast ads that do not give audiences a brief summary of a drug’s risks. No ads included a major statement. There were approximately 7.18 risk facts for every benefit fact. Most of the risks (98.85%, 1292/1307) were presented in the scroll portion of the ad, whereas most of the benefits (66.5%, 121/182) were presented in the main part of the ad. Out of 1307 risk facts, 1292 were qualitative and 15 were quantitative. Out of 182 benefit facts, 181 were qualitative and 1 was quantitative. The majority of ads showed neutral images during the disclosure of benefit and risk facts. Only 9% (6/68) of the ads displayed positive images and none displayed negative images when presenting risks facts. When benefit facts were being presented, 7% (5/68) showed only positive images. No ads showed negative images when the benefit facts were being presented.

**Conclusions:**

In the face of ambiguous regulatory guidelines for online banner promotion, drug companies appear to make an attempt to adapt to regulatory guidelines designed for traditional media. However, banner ads use various techniques of presentation to present the advertised drug in the best possible light. The FDA should formalize requirements that drug companies provide a brief summary and include multiple forms of adequate provision in banner ads.

## Introduction

### Background

 Direct-to-consumer advertising (DTCA) of prescription drugs has skyrocketed since the late 1990s. From 1996 to 2005, DTCA expenditures grew from US $985 million to more than US $4.2 billion, a 330% rise [1]. This rise was due to regulatory changes made by the Food and Drug Administration (FDA) in the late 1990s that clarified and relaxed the existing DTCA guidelines. Prior to 1997, all consumer-directed prescription drug ads broadcasted were required to provide a brief summary of a drug’s risks and side effects to consumers. Brief summaries were often lengthy, and although it was straightforward to disclose the content of a brief summary in print media, this was more difficult in a broadcast advertisement. In 1999, the FDA released a final guidance [2] allowing drug manufacturers advertising on television and radio to include a “major statement” identifying the drug’s major risks in lieu of the brief summary. This statement, which must be spoken, is relevant only to broadcast ads. The FDA also requires broadcast advertisers to provide “adequate provision,” which the FDA defines as “an alternative way for drug companies to provide risk information about a drug in a broadcast ad” [3] in lieu of the brief summaries.

The new regulations detail appropriate ways drug companies can provide adequate provision. These include providing consumers with (1) a toll-free telephone number they can call to listen to a reading of the brief summary, (2) a webpage address where they can access product information, (3) a statement that encourages consumers to consult a health care professional for more information about a drug, or (4) an alternative mechanism, such as a print resource, to access the brief summary relating to the drug product. The FDA also reiterated certain existing regulations that stipulated all promotion must present a “fair balance” between information relating to the drug’s risks and information relating to its benefits. With these changes, drug companies could broadcast product claim ads or ads that make benefit and risk claims for a prescription drug without listing numerous medical definitions and studies as stipulated by the previous regulations.

Although total DTCA expenditures from 2005 to 2010 declined slightly, the subcategory of online promotion experienced a significant increase in expenditures [4,5]. It is unclear whether the current DTCA guidelines for print, radio, and television media or the recent guidelines for select forms of Internet advertising apply to online banner advertising. Many scholars are concerned that policymakers are not doing enough to regulate the online prescription drug advertising environment [6-8]. A lack of detailed policies that target the various forms of online advertising may lead drug companies to fail to disclose adequate information about the risks and benefits of prescription drugs to consumers. This study investigates the degree to which one common form of Internet advertising, banner ads, comply with existing regulations for print and television ads, and the adequate provision and fair balance requirements, in particular.

### Prior Work

Previous research has analyzed the risk and benefit content of prescription drug ads. In particular, studies have documented DTCA deficiencies, including (1) the use of false or misleading claims [9,10], (2) not giving adequate attention to risk factors or causes of a condition [11], and (3) describing benefits in vague and ambiguous terms without citing scientific studies [11,12]. However, studies have shown that, in general, pharmaceutical drug ads tend to comply with the adequate provision and major statement requirements [13-16].

Regarding fair balance in television and print ads, most research has found ads to be deficient. Many DTCA studies have found that ads play up benefits and give short shrift to risks [11,13,15,17-22]. For example, one study revealed that, on average, audiences were given a third less time to absorb the risks compared to the benefits; moreover, 83% of ads included in the study presented risks in a single continuous segment rather than at various points throughout the ad [13]. One exception is a study of television ads that concluded that fair balance was reached given that prescription drug ads had similar numbers of benefit and risk statements per 30 seconds of ad time [16]. This study’s discrepant findings can be explained by the manner in which it measured fair balance. Studies that compare counts of references to side effects, contraindications, and effectiveness are more likely to conclude fair balance is achieved than studies that measure the way information is presented, such as a study examining whether information was presented in a continuous segment as well as the types of images shown during risk and benefit information presentation [13]. The authors of these studies are more likely to conclude that although the sample of ads contain more information about risks than benefits, the ads present information in such a way as to downplay those risks relative to the benefits. The FDA does not offer detailed guidelines on how to achieve fair balance, but it does advise that information about side effects and effectiveness should be of comparable “prominence and readability” [2], which suggests that advertisers should pay attention to the form and context in which information is being presented.

Online advertising comes in different forms, including banner ads, whole websites, social media, coupons, and email promotions, among others. Conducting research on banner advertising is important because although research shows that online ads are ignored by website viewers, this does not mean Internet promotion does not have an effect on consumers [23,24]. Research has found that banner ads can have an effect on consumers through unconscious cognitive processes [25,26]. One manipulated experiment found that both a group of participants who were directed to look at banner ads on websites and a group whose attention was not directed to banner ads developed more favorable attitudes toward the advertised brand compared to a control group [26].

Although traditional media remains the most common form of DTCA promotion, Internet promotion has accounted for an increasing proportion of all DTCA since the early 2000s [4]. There are now many studies that focus on online prescription drug advertising. However, these studies have mostly focused on investigating topics such as the prevalence and nature of prescription drug advertising via social media [7,27-29], the stock market reaction to noncompliance [30], and commentaries about the political, ethical, and/or legal problems associated with various forms of online prescription drug promotion [6,31-35]. One recent review of the FDA Warning Letters and Notice of Violation Letters sent to pharmaceutical companies for online prescription drug ads in violation found that the majority of violations concerned a lack of risk information and/or misrepresentation of the drug’s efficacy [36]. However, this study did not directly investigate the risk and benefit content of online advertising promotion, and there are very few such studies. Moreover, the few studies that do examine fair balance and adequate provision in online DTCA are dated and inconsistent as to whether drug manufacturers present risks and benefits in a fair and balanced way. For example, a study of 90 pharmaceutical company drug websites found that most websites meet fair balance and adequate provision guidelines and concluded that websites were superior to print advertisements because they offered consumers a greater degree of medical and drug information [14,37]. However, other studies of pharmaceutical company websites found that although the websites contain risk and benefit information, drug companies present this information so as to highlight benefits and downplay risks, thus not meeting the FDA guidelines [38,39].

### Study Aims

**Figure 1:**
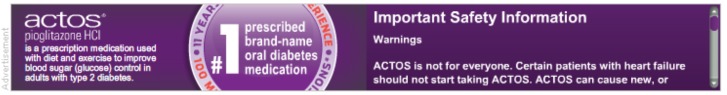
Actos banner advertisement.

The purpose of this study is to investigate the degree to which one form of online prescription drug promotion, Internet banner ads, comply with adequate provision and fair balance FDA guidelines (see Figure 1 and Multimedia Appendices 1A-E for examples of DTCA banner ads). Banner ads are visuals that are placed on a hosting website containing promotional information and often include hyperlinks to the sponsoring website that contains more detailed information about a product. Banner ads were chosen for analysis over other forms of Internet promotion for 2 reasons. First, banner ads continue to be a popular form of online advertising. In 2014, banner ad revenues were US $8 billion, a 16% share of online ad dollars [40]. Second, there has not been a DTCA content analysis of banner ads to date despite a need for research on this media. The focus of this study is on the adequate provision and fair balance guidelines because these are the central requirements for DTCA, although it is unclear whether the adequate provision guideline applies to banner ads. Given that these ads are typically small, it may be infeasible to display a lengthy brief summary. Therefore, this research investigates whether ads disclose the brief summary or not and, if not, whether they make adequate provision for the prescribing information. The ad’s hyperlinked page, which almost always directs the user to the drug company website, was not chosen for analysis because the FDA does not uphold the “one-click rule,” which states that an online prescription drug ad can mention the brand name and the benefits without including all or any of the major side effects as long as fair balance is just one click away.

The research questions of this study are as follows:

1. Do banner ads include brief summaries, major statements, or otherwise make adequate provision for prescribing information?
2. Do banner ads achieve fair balance as measured by the ratio of risk facts to benefit facts?
3. Do banner ads achieve fair balance as measured by the presentation of risk and benefit information in banner ads?

## Methods

### Data Collection

Product-specific ads were selected over a 1-month period during April 2011. Because this project was concerned with ads that reach a broad audience rather than an information-seeking audience specifically, the sampling frame for online ads included websites geared to a broad audience. During preliminary testing, however, it was discovered that random browsing on popular websites made it difficult to locate prescription drug ads. This is likely due to drug companies’ efforts to reach their target audiences by advertising heavily on websites related to health. Therefore, to increase the likelihood of locating drug ads, health-related websites were selected for monitoring.

Google’s “List of the Most Visited Webpages” [41] for 2011 was used to identify the websites with high traffic volume. This list is based on unique visitors (users), as measured by Ad Planner, and includes information about the site category, the number of unique visitors, whether the site does or does not accept advertising, and the region(s) of the world where the website is popular. The top 10 Web portals and news websites found on this list were chosen. Web portals were chosen because they are highly trafficked general interest sites that serve as gateways to other areas of the Internet. Because there were a limited number of Web portals on Google’s list, news websites were also included in the sampling frame.

Also, eBizMBA’s “Top 15 Most Popular Health Websites” [42] for 2011 was used to select the health websites. eBizMBA compiled this list based on the average of each website’s Alexa Global Traffic Rank, and US traffic rankings from both Compete and Quantcast. From this list, the top 10 websites that accept advertising funding from drug companies were monitored daily. Table 1 provides a summary of the monitored websites. A total of 68 unique banner ads were gathered. This is a sample size similar to those of other studies of online prescription drug promotion [17,37,39].

**Table 1:** Summary of monitored websites (N=20).

Website		Website type	US rank^a^	Unique visitors per month^a^
**Health-related**				
	Yahoo! Health	Health	209	21,500,000
	WebMD	Health	247	19,500,000
	MedicineNet.com	Health	563	10,500,000
	Drugs.com	Health	871	6,000,000
	Everyday Health	Health	969	5,700,000
	WrongDiagnosis.com	Health	1203	4,700,000
	MedHelp	Health	1243	4,600,000
	RightHealth	Health	1590	4,150,000
	Wellsphere	Health	1726	3,900,000
	RxList	Health	2601	2,400,000
**Non–health-related**				
	Yahoo!	Web portal	2	110,000,000
	MSN	Web portal	5	450,000,000
	The Walt Disney Company	Web portal	13	81,000,000
	AOL	Web portal	16	72,000,000
	CNN	News	20	50,000,000
	The New York Times	News	34	12,000,000
	Fox News	News	50	8,200,000
	The Huffington Post	News	51	7,500,000
	The Washington Post	News	72	5,600,000
	The Wall Street Journal	News	80	5,600,000

^a^ Based on Google’s “List of the Most Visited Webpages” for 2011 [41].

A preliminary examination of the websites revealed that almost all banner ads were animated at some point in the ad’s duration and included a scroll feature containing a brief summary of indications and side effects (see Figure 1 and Multimedia Appendices 1,A, B, and E for examples of the scroll feature). In order to record a real-time account of animated ads, TechSmith’s Camtasia Studio 7.0, a screen video recorder, was used to provide a timed account of every action that took place on the screen during ad play. For all monitored websites, 3 screen recordings—one in the morning, one in the early afternoon, and one in the evening—of each website were taken daily for a 1-month period. Screen recordings were taken at 3 different times of day to attempt to capture any variation in the types of ads that were displayed at different times of the day. The browser’s cache was cleaned after recording each advertisement so as not to bias the sample based on previous browsing history. Ads that did not include an automatic scroll required the user to manually scroll within the ad to view additional information, such as risk and benefit information. The data were stored and managed in NVivo 10. A document composed of unitized statements, defined as complete assertions made or images displayed, was created for each ad. These documents were imported into NVivo along with an internal link to view the ad.

### Coding Scheme

A coding sheet (see Multimedia Appendix 2) was developed based on (1) the FDA’s guidance documents for consumer-directed prescription drug promotion on television and 2) previous DTCA content analyses [13,16,21]. The following descriptive information was gathered from all ads: the drug’s brand name, the condition(s) the drug was promoted to treat, whether the ad described the condition the drug was promoted to treat, and any mention of the causes of or risk factors for the condition(s). The presence of a scroll and whether the scroll was automatic or manual (requiring the viewer to move the scroll button) was also documented. Also, all links were clicked on to determine whether they were active.

The brief summary was operationalized according to FDA brief summary guidelines, which require manufacturers to provide “all the risks listed in the drug’s ‘prescribing information’ and at least one FDA-approved use of the drug” [3]. Although it was considered unlikely for major statements to be included in banner ads because major statements must be verbal and banner ads usually do not include audio, the presence of a major statement was documented. Adequate provision was measured by documenting the ad’s reference to one of the 4 forms of adequate provision accepted by the FDA [13,16].

Fair balance was measured both in terms of the number of benefit and risk facts present in the ads and in terms of the presentation of the risk and benefit information. The number of benefit facts and risks facts were counted and a ratio of benefit to risk facts was calculated. Following the FDA [3] and previous research [16], a *benefit fact* was defined as any purported positive outcome from taking a drug and a *risk fact* was defined as any possible negative outcome from taking a drug. Neutral facts not related to risks or benefits (eg, identifying the generic name of a brand name drug or directives for how to use the drug) were not coded because the study was interested in analyzing the benefit and risk content of banner ads, which is how the FDA defines fair balance. The author referred to the FDA product label to determine the risks and indications of the promoted drug.

The presentation of risk and benefit information was analyzed because of research showing that contextual elements matter for how audiences absorb factual information in ads [13,16,38,39]. This study concentrated on 3 aspects of presentation identified in previous research [13]. First, the study documented whether qualitative or quantitative terms were used to describe benefits and risks. Qualitative terms included such words as “low,” “high,” and “reduce,” whereas quantitative terms used numbers to describe risks and benefits. Each benefit and risk fact was categorized as qualitative or quantitative. Second, the visual images shown during the presentation of risk and benefit facts were assessed. Images were grouped into 2 broad categories: positive and negative. Images were considered positive (see Multimedia Appendices 1A and B for examples) or negative if the visual scenes or actors evoked positive or negative feelings or were positive or negative portrayals. Finally, whether the benefits and risks were presented in the main part of the ad versus in the scroll box was assessed, and the percentage of risks and benefits presented in the scroll and main portions of the ad were calculated. The risk-to-benefit ratio for facts presented in the scroll and main portions of the ad was also calculated.

At the beginning of the coding process, an independent researcher was recruited to code 5 ads in order to pilot-test the coding scheme. The codebook was modified to resolve any discrepancies that came to light during the pilot test. For time and cost reasons, the author then completed the rest of the coding independently. At the end of the coding process, the researcher involved in the pilot test of the coding scheme coded a random sample of 17 ads (one-quarter of the sample) to test for intercoder reliability. Kappa coefficients were computed for the following variables related to the research questions: brief summary, major statement, adequate provision, risk and benefit facts, qualitative and quantitative language, positive and negative images, and the presence of risks and benefits in the main part of the ad versus in the scroll box. Intercoder agreement for the presence and absence of the brief summaries, major statements, and the different forms of adequate provision was perfect at κ=1. The kappa coefficients for the remaining variables ranged from .55 to .61, which can be regarded as moderate to substantial agreement [43]. See Table 2 for the kappa coefficients for each category.

**Table 2:** Kappa coefficients for intercoder reliability.

Variable		Kappa
Brief summaries		1
Major statements		1
**Adequate provision**		
	Doctor reference	1
	Print ad reference	1
	Website address	1
	Toll-free number	1
	Prescribing information	1
	Medication guide	1
**Benefit and risk information**		
	All benefit facts	.61
	Qualitative benefit facts	.55
	Quantitative benefit facts	.58
	Benefit facts in main portion of ad	.60
	Benefit facts in scroll portion of ad	.61
	Positive images display (benefits)	.61
	Negative images display (benefits)	.59
	All risk facts	.59
	Qualitative risk facts	.56
	Quantitative risk facts	.59
	Risk facts in main portion of ad	.61
	Risk facts in scroll portion of ad	.59
	Positive images display (risks)	.60
	Negative images display (risks)	.59

## Results

### Descriptive Statistics

**Figure 2:**
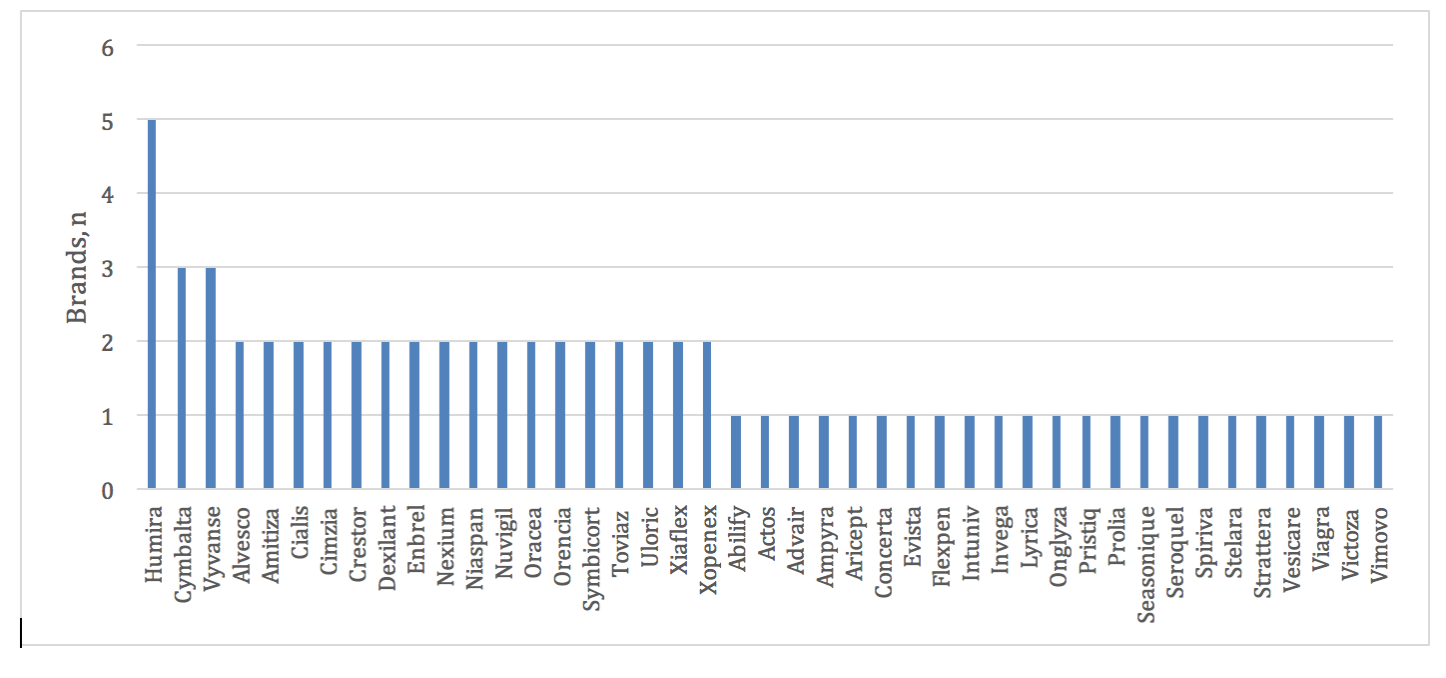
Number of brands represented in sample.

**Figure 3:**
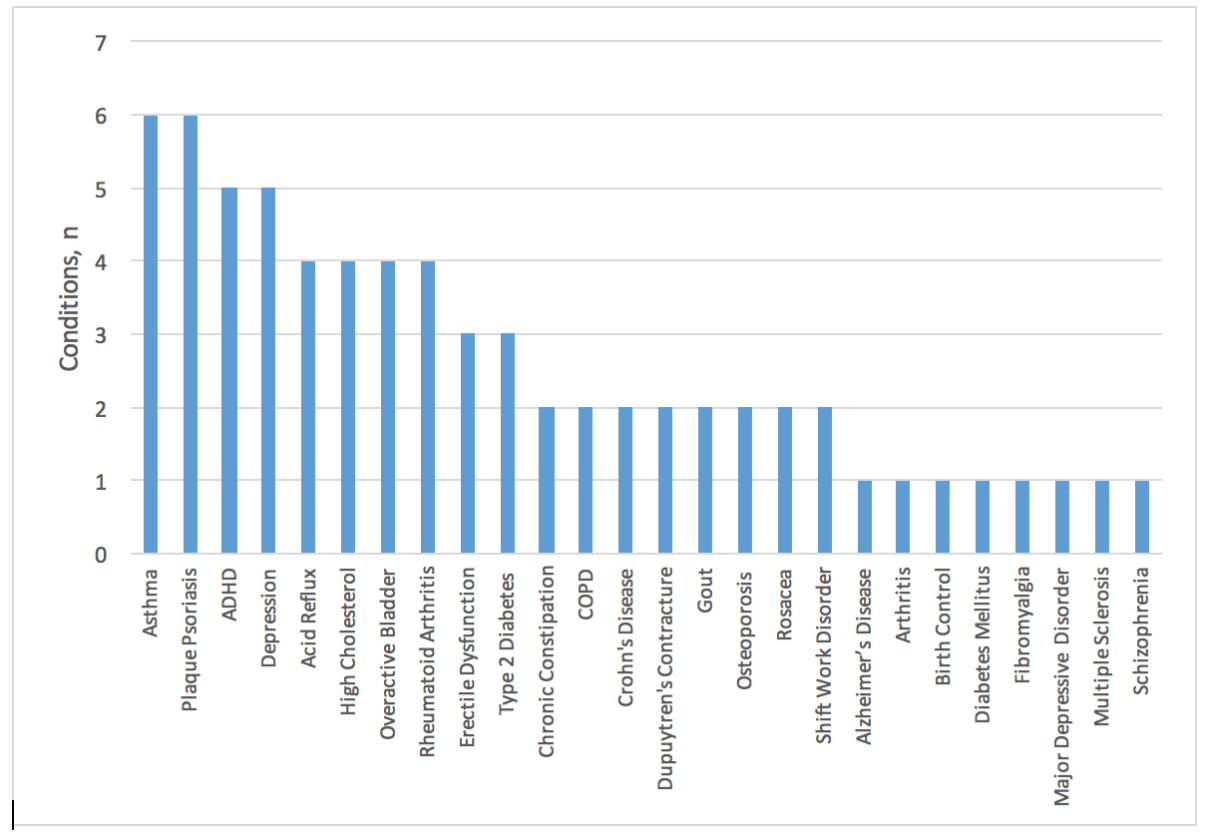
Number of conditions represented in sample.

A total of 212 (including repeat) ads and 68 unique banner ads were gathered; 43 brand names were represented in the sample (see Figure 2). Of these, the most common were Humira with 5 unique ads and Cymbalta and Vyvanse with 3 unique ads each. Figure 3 shows the frequency of health conditions targeted in the sample. In all, 26 conditions were represented in the sample, the most common being asthma, plaque psoriasis, attention-deficit/hyperactivity disorder (ADHD), and depression. Table 3 provides a summary of the condition-related descriptive statistics and the scroll type used to display the brief summary. Most ads did not go to great lengths to describe a condition. Approximately 29% (20/68) of all ads described the condition the drug was designed to treat. A little over 7% (5/68) of ads mentioned the causes of a condition and no ads (0%, 0/68) mentioned the risk factors for a condition. All banner ads (100%, 68/68) had a scroll box within the ad that contained the brief summary (see the “Important Safety Information” portions of Figure 1 and Multimedia Appendices 1A, B, D, and E for examples of brief summaries). Close to 46% (31/68) of ads had an automatic scroll and 54% (37/68) required the user to scroll. All links in all ads were active. Every banner included a hyperlink on the main part of the ad directing the user to the drug company webpage for the pharmaceutical drug in question (see the “Learn more” hyperlink in Multimedia Appendix 1C for an example). Occasionally, the main ad also included separate links to coupons (eg, the free trial offer shown in Multimedia Appendix 1D) or condition information. These webpages were always hosted on the drug company website.

### Brief Summary, Major Statement, and Adequate Provision

All banner ads included the brief summary in the scroll box. No ads included a major statement. Because all ads included a brief summary, a major statement was not required under DTCA broadcast regulations. In addition to the brief summary, all ads except one included links to the drug’s prescribing information (see Table 3; see the hyperlink in Multimedia Appendix 1B entitled “Prescribing Information” for an example). These links provided information about a drug’s indications, contraindications, and risks. Many ads (53%, 36/68) also included links to the drug’s medication guide (see the hyperlink in Multimedia Appendix 1B entitled “Medication Guide” for an example), which the FDA requires for certain prescription drugs and drug classes with particularly serious adverse effects, in addition to prescribing information. It should be noted that 46% (31/68) of banner ads were for drugs with black box warnings. Some common drugs in the sample with black box warnings were indicated to treat Crohn’s disease, ADHD, and depression. Although some drugs with black box warnings required medication guides and some did not, all the ads in the sample for drugs with medication guides disclosed links to these guides.

**Table 3:** Number of ads containing prescribing information and medication guides and different forms of adequate provision (N=68).

Variable		Total, n (%)
**Type of guide**		
	Prescribing information only	31 (46)
	Medication guide only	0 (0)
	Both prescribing information and medication guide	36 (53)
	Neither prescribing information nor medication guide	1 (1)
**Type of adequate provision**		
	Doctor reference	68 (100)
	Print ad reference	0 (0)
	Website address	68 (100)
	Toll-free number	0 (0)

If FDA broadcast ad regulations apply to banner ads, the inclusion of a brief summary in an ad would obviate the need to include adequate provision for the brief summary. However, all ads included some form of provision with additional information about the drug (see Table 3). All banner ads included both a doctor reference and a URL for full access to product information. No ads included a phone number or a reference to a print ad. In addition to the usual forms of adequate provision, 63% (43/68) of banner ads provided the audience with the contact information for MedWatch, the FDA’s voluntary adverse event reporting program. According to the FDA Amendments Act of 2007, print (but not broadcast) ads must include a statement referring the audience to the MedWatch program (see the “Important Safety Information” section of Multimedia Appendix 1E for an example of a reference to MedWatch).

### Fair Balance

Table 4 presents information on the number of risk and benefits facts and the risk/benefit ratios. There were 1307 risk facts in the total sample, with a mean 19.22 (SD 9.91) risk facts per total number of advertisements compared to 182 benefit facts in the total sample, with mean 2.68 (SD 1.32) per total number of advertisements. There were approximately 7.18 risk facts for every benefit fact. Most of the risks (98.85%, 1292/1307) were presented in the scroll portion of the ad, whereas most of the benefits (66.5%, 121/182) were presented in the main portion of the ad. The risk/benefit ratio was 21.18 for facts presented in the scroll portion of the ad and 0.12 for those presented in the main portion of the ad.

**Table 4:** Risk and benefit facts in ads.

Variable		n	Facts, mean (SD)^a^	Ratio of risk facts to benefit facts
**Risk facts**		1307	19.22 (9.91)	7.18
	Main portion of ad	15	0.22 (1.08)	0.12
	Scroll portion of ad	1292	19.00 (9.85)	21.18
**Benefit facts**		182	2.68 (1.32)	
	Main portion of ad	121	1.78 (1.12)	
	Scroll portion of ad	61	0.90 (1.25)	

^a^ Per total number of advertisements.

Table 5 provides information on the presentation of risks and benefits in ads. Of the 1307 risk facts, 1292 were qualitative and 15 were quantitative. All ads (100%, 68/68) contained qualitative risk facts and 6% (4/68) contained quantitative risk facts. When presenting risks facts, 9% (6/68) displayed positive images and no ads displayed negative images. Thus, 91% (62/68) relied only on neutral image content when presenting risk facts.

**Table 5:** Presentation of benefit and risk information.

Variable			Ads, n (%)	Facts, n	Facts, mean (SD)^a^
**Qualitative or quantitative presentation**					
	**Risk**				
		Qualitative	68 (100)	1292	19.00 (9.97)
		Quantitative	4 (6)	15	0.22 (1.05)
	**Benefit**				
		Qualitative	68 (100)	181	2.66 (1.30)
		Quantitative	1 (1)	1	0.01 (0.12)
**Image presentation**					
	**Risk**				
		Positive	6 (9)	n/a^b^	
		Negative	0 (0)	n/a^b^	
		Neutral	62 (91)	n/a^b^	
	**Benefit**				
		Positive	5 (7)	n/a^b^	
		Negative	0 (0)	n/a^b^	
		Neutral	63 (93)	n/a^b^	

^a^ Per total number of advertisements.

^b^ The presence/absence of images rather than the number of images was coded.

Of the 182 benefit facts, 181 were qualitative and only one was quantitative. All ads contained qualitative benefit facts and only one ad contained a quantitative benefit fact. The majority of ads (93%, 63/68) displayed neutral images when presenting benefit facts; 7% (5/68) of ads showed only positive images and no ad (0%, 0/68) displayed negative images when the benefit facts were being presented.

## Discussion

### Principal Findings

This study supports prior content analyses of prescription drug ads that find that ads largely comply with the adequate provision and major statement broadcast requirements [13-16]. Although ads did not use a toll-free phone number and print ads as provision, they disclosed other forms of provision that may be helpful to patients. The banner ads abide by the print ad regulation requiring the inclusion of the MedWatch statement, which may be helpful to the public by informing them of this reporting mechanism. Banner ads did not achieve fair balance in terms of the ratio of risk to benefit facts in the main portion of the ad. Regarding presentation, the findings support prior research that concludes that ads highlight information about benefits more than information about risks [11,13,15,17-22].

In the face of ambiguous regulatory guidelines for online banner promotion, drug companies appear to make an attempt to adapt to regulatory guidelines designed for traditional media. However, banner ads use various techniques of presentation to present the advertised drug in the best possible light. First, ads display risks in a small box embedded in the ad. Because most ads either require the user to manually scroll or have a rapid automatic scroll, user engagement is often required to access risk information. Second, ads are more likely to show positive than negative images when presenting either risk or benefit information. Third, ads use qualitative as opposed to quantitative terms to describe risks and benefits. It is debatable whether the reliance on qualitative descriptions is good or bad for audiences. The FDA has recently acknowledged that the use of lay terms is preferable over an overreliance on medical terminology because consumers can more easily retain information communicated in consumer-friendly terms [44].

### Policy Significance of Study

A review of the FDA Warning Letters that were sent to pharmaceutical companies in violation of FDA guidelines revealed that the FDA has sent pharmaceutical companies only 3 letters for banner advertisements in violation of FDA protocol. The FDA first sent an enforcement letter to a firm for a banner ad in violation in 1998. This letter informed GD Searle & Co that their website banner for Daypro failed to achieve fair balance by not providing any information related to side effects or contraindications [45]. The FDA sent a different letter to Novartis in 2008 for the same fair balance violation, claiming that 8 online banner ads for the drug Diovan presented only efficacy claims and omitted all risk information [46]. A third letter sent in 2009 to GlaxoSmithKline for 5 banner ads for Treximet reveals more about the FDA’s regulatory approach toward banner ads [47]. According to this letter, the Treximet ads minimized serious risks by devoting most ad space to text and visual presentations of the drug’s effectiveness while underrepresenting risks by placing this information in an automatic scroll in a small slice of the banner ad. The letter stated: “Unlike the efficacy claims in the banners, the risk is presented without any signals or other attention-grabbing devices to alert readers that this is important information about the drug” (pg. 3) [47]. As revealed by this study’s findings, the troublesome design elements of the Treximet ads are still common, showing that marketers have not altered their design of banner ads to address the concerns about fair balance communicated in the 2009 Treximet enforcement letter.

The FDA has responded to critics repeatedly calling for the FDA to directly address Internet advertising of medical products [48-51]. The FDA facilitated a public hearing in November 2009 to discuss the topic. At this meeting, many pharmaceutical industry representatives supported the “one-click rule.” However, the enforcement letters sent by the FDA to companies displaying sponsored links on Google where audiences could access full product information in just one click indicate it does not support the one-click rule.

Subsequent to the November 2009 meeting, the FDA released a series of guidelines that are relevant to Internet promotion and social media. The first came in 2011 [52] and provided guidance to pharmaceutical companies on how to respond to unsolicited requests for off-label information about prescription drugs and medical devices. Many firms receive such requests through firm-controlled product websites, discussion boards, chat rooms, and other electronic forums. The guidance says that the pharmaceutical company’s public response to unsolicited requests for off-label information should be limited to delivering the contact information of the medical or scientific personnel or department, should ensure that responses are not promotional in nature, and should not include any details regarding off-label information. The firm can only provide individuals with a detailed response about off-label uses privately, not publically.

A few years later, in June 2014, the FDA released 2 additional draft guidelines specifically devoted to the Internet and social media. The first document informs drug and device manufacturers how to correct misinformation on third-party websites about their products [53]. This guidance only applies to firms that are not responsible for the product communication that contains misinformation and, thus, does not apply to DTCA banner ads. The second targeted social media promotion with character space limitations, such as Twitter or sponsored links on search engines [54]. This guidance requires promotion via character-limited media to accurately present risk information along with benefit information. This is not possible in extremely limited message space, thus barring promotional activities in these media. The second guidance does not apply to online Web banners because the FDA deemed that this type of social media platform does not impose the same character space limitations as other forms of social media, such as online paid search ads and Twitter.

The new guidelines do not support the existence of a one-click rule. On May 20, 2015, Representative Billy Long of Missouri introduced Bill HR 2479 [55] to the House of Representatives that would make the one-click rule law. The bill would enable firms to engage in promotional activities in character-limited applications by regarding hyperlinked information in such media “as if the information appeared in introductory information” (ie, the original character-limited text). If signed into law, this bill would require the FDA to review and revise all guidance within 6 months and publish final regulations related to matters described in HR 2479 within 18 months. The future of this bill remains to be seen.

### Limitations

This study has several limitations. The data are cross-sectional and, thus, represent only a snapshot in time. The Internet is a constantly changing medium and marketers will devise new innovations to utilize its functionalities in ways that can either benefit or hinder the public’s understanding of the uses and risks of prescription drugs. The data were gathered in 2011; thus, this is not an up-to-date representation of the state of prescription drug banner advertising. However, a nonsystematic review of banner ads using Moat, an advertising search engine, revealed that banner ads do not differ much now in format or content compared to 2011 (see Multimedia Appendix 3 which displays screenshots of ads in the sample and newer ads [as found in Moat] for select drugs). Additionally, it is important to document how banner ads have changed over time. Future studies of banner DTCA can compare the quality and presentation of risk and benefit information to this study’s findings. The sample size may be seen by some as small; however, it is similar to other DTCA content analyses [17,37,39]. Also, although an outside researcher was involved in testing the coding scheme and determining intercoder reliability, this was done for only a sample of the ads and not the full sample. In addition, some researchers express concern with reliabilities that range in the area of .5 to .6. [56,57]. Another limitation concerns the distinction between quantitative/qualitative language and positive/negative images, which are broad dichotomies and do not represent the full spectrum of how language and image content can be communicated. Finally, the search strategy was constrained by the fact that website tracking determines the advertisements that websites display to consumers. This makes it difficult to gather a truly representative sample of prescription drug banner ads.

### Conclusions and Future Research

Although helpful in regulating many problems that drug companies confront in the Internet social media climate, the new guidance that pertains to electronic media does not provide much direction to firms seeking to generate banner ads in compliance with regulations. Given the constantly changing nature of the Internet, it is difficult to create guidelines for each specific medium. However, the FDA should nonetheless continually construct new guidance and revise past guidance so as to provide direction to the industry and identify instances of malfeasance. The United States is only one of two developed countries in the world to allow DTCA (the other is New Zealand). If it is to continue to permit DTCA, it is the FDA’s responsibility to strictly monitor and enforce existing regulatory principles for the protection of patients and this involves keeping up with the changing media climate. Given the ubiquity of the Internet, the quality of information in the United States could impact patients outside the United States as well.

For banner ads in particular, the FDA should resolve the apparent inconsistency with its statements in the GlaxoSmithKline Treximet letter and its subsequent lack of action on banner ads with the very same features as the Treximet ads. It should consider formalizing a requirement that drug companies disclose the brief summary in banner ads or, if not, identify the appropriate forms of adequate provision. If the FDA were to make the one-click rule law, studies of banner ads would need to review the hyperlinks on banner ads to determine fair balance.

This is the first content analysis of banner ads. Future research on banner ads should investigate (1) how audiences receive information in the typical design format of DTCA banner ads and (2) how banner ads can be altered—if at all—so as to achieve optimal audience understanding of the risks and benefits of medical products. Research on other types of Internet promotion, such as email advertising and online forums on drug company websites, is also necessary to advise the FDA on how best to deliver accurate information about prescription drugs to consumers. Evidence-based research can provide insight as to how the Internet’s functionalities can be utilized to better communicate risk and benefit information to consumers. The interactive nature of the Internet allows for features not possible with traditional media, such as pop-up windows, links to more information, and embedded videos. Thus, viewers learning about a prescription drug for the first time on the Internet can quickly access additional information. Future studies should also assess the strategies the industry uses to target certain patient groups and demographic populations via online marketing.

## Appendix

Multimedia Appendix 1 (A) Cialis banner advertisement. (B) Evista banner advertisement. (C) Niaspan banner advertisement. (D) Abilify banner advertisement. (E) Niaspan banner advertisement.

Multimedia Appendix 2 Coding scheme.

Multimedia Appendix 3 Screenshots of original ads in sample and newer ads for select drugs.

## Abbreviations

ADHD: attention-deficit/hyperactivity disorder
DTCA: direct-to-consumer advertising
FDA: Food and Drug Administration
