# Supplementary material for: Fair Balance and Adequate Provision in Direct-to-Consumer Prescription Drug Online Banner Advertisements: A Content Analysis
Source: J Med Internet Res. 2016 Feb 18;18(2):e33. doi: 10.2196/jmir.5182 (PMC4777882; doi:10.2196/jmir.5182)

(A) Cialis banner advertisement.

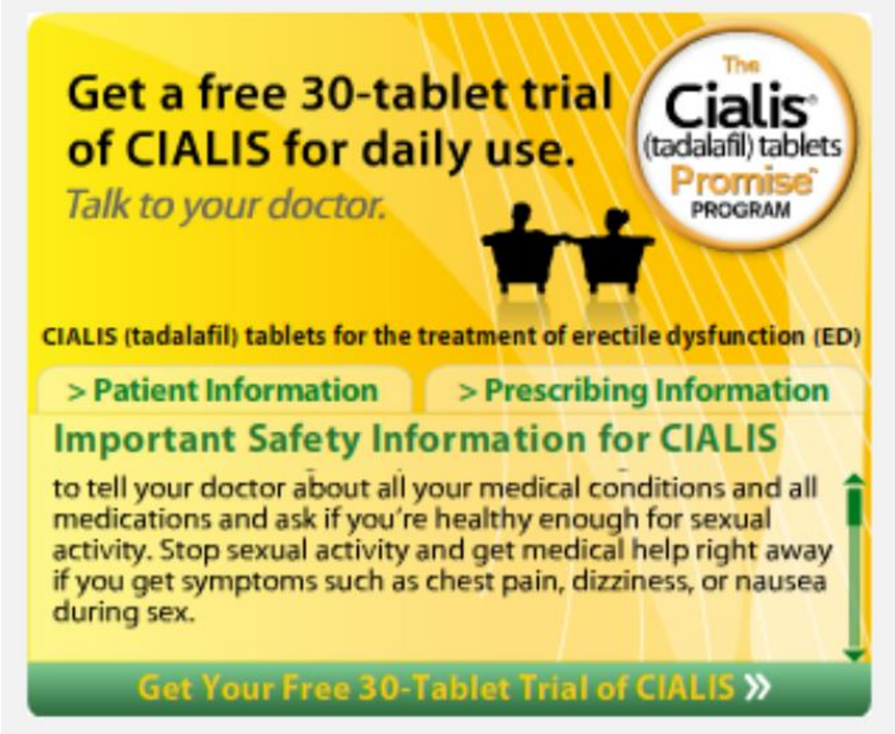

(B) Evista banner advertisement.

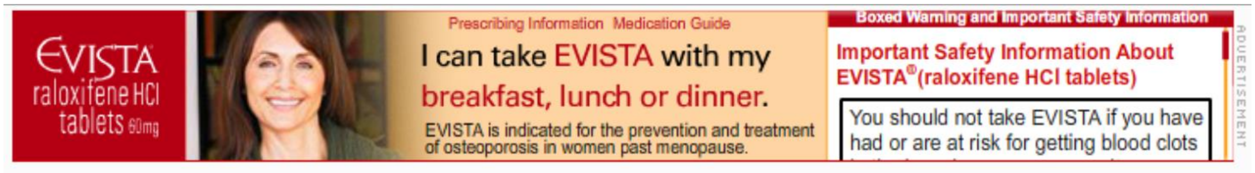

(C) Niaspan banner advertisement.

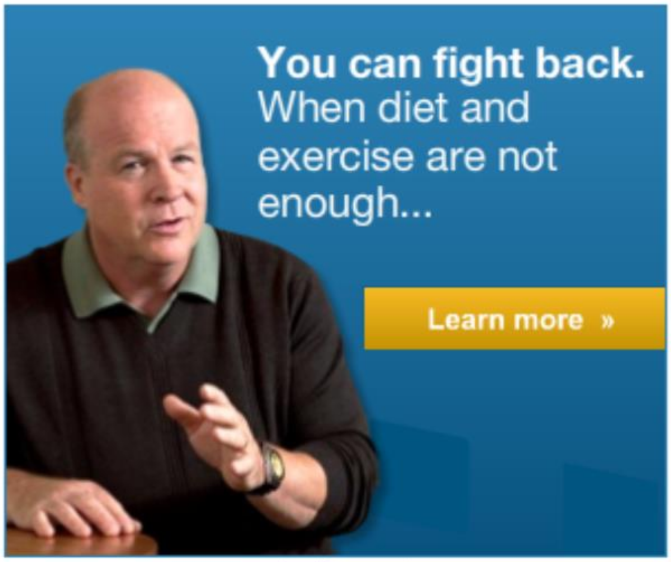

advertisement

(D) Abilify banner advertisement.

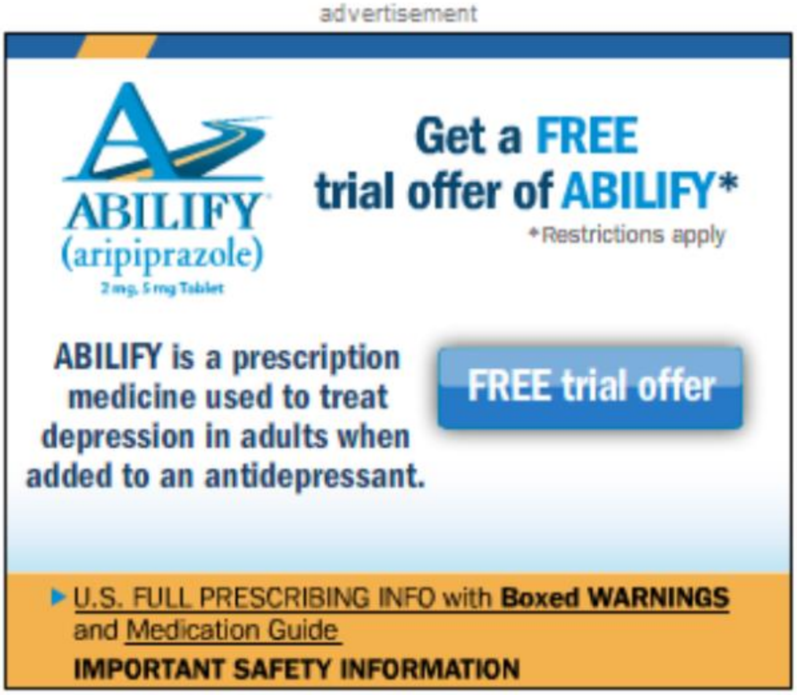

(E) Niaspan banner advertisement.

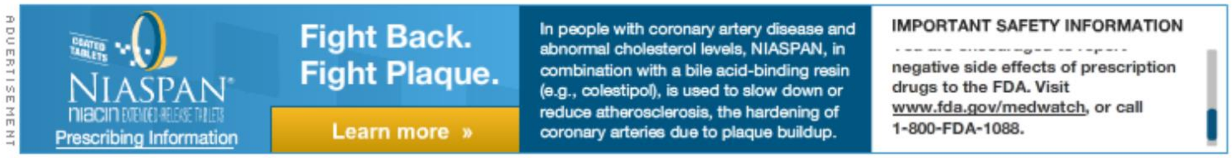

Supplement: Multimedia Appendix 1 [file jmir_v18i2e33_app1.pdf]
