# Supplementary material for: Fair Balance and Adequate Provision in Direct-to-Consumer Prescription Drug Online Banner Advertisements: A Content Analysis
Source: J Med Internet Res. 2016 Feb 18;18(2):e33. doi: 10.2196/jmir.5182 (PMC4777882; doi:10.2196/jmir.5182)
Supplement: Multimedia Appendix 2 [file jmir_v18i2e33_app2.pdf]

Multimedia Appendix 4. Coding Scheme

Descriptive statistics
Condition description
Condition causes
Risk factors
Automatic scroll type
Manual scroll type

Brief summary
Major statement

Adequate provision
Doctor reference
Print ad reference
Website address
Toll-free number
Prescribing information
Medication Guide

Benefit and risk information
All benefit facts
Qualitative benefit facts
Quantitative benefit facts
Benefit facts in main portion of ad
Benefit facts in scroll portion of ad
Positive images display (benefits)
Negative images display (benefits)

All risk facts
Qualitative risk facts
Quantitative risk facts
Risk facts in main portion of ad
Risk facts in scroll portion of ad
Positive images display (risks)
Negative images display (risks)
