# Supplementary material for: Fair Balance and Adequate Provision in Direct-to-Consumer Prescription Drug Online Banner Advertisements: A Content Analysis
Source: J Med Internet Res. 2016 Feb 18;18(2):e33. doi: 10.2196/jmir.5182 (PMC4777882; doi:10.2196/jmir.5182)

Multimedia Appendix 7. Screenshots of original ads in sample and newer ads for select drugs

Humira

2011 version:

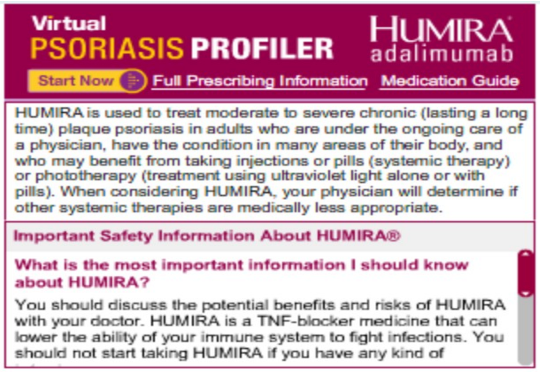

2015 version:

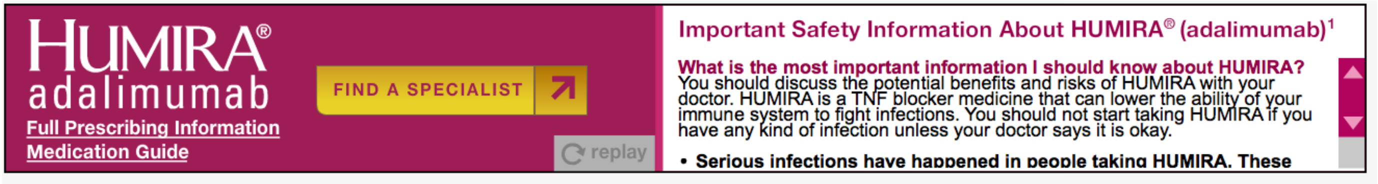

Cymbalta

2011 version:

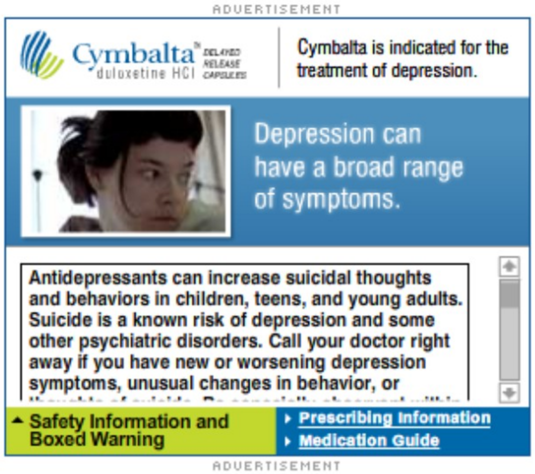

2013 version:

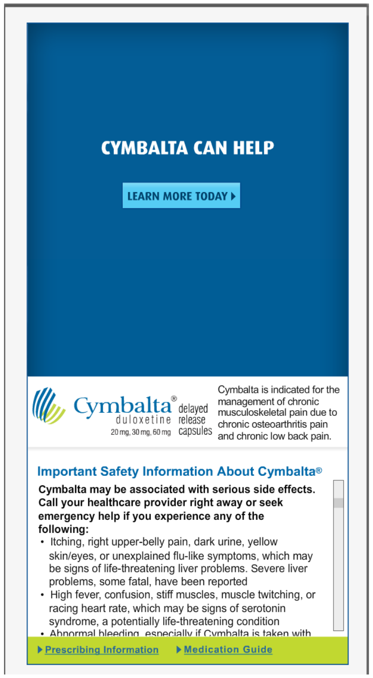

Vyvanse

2011 version:

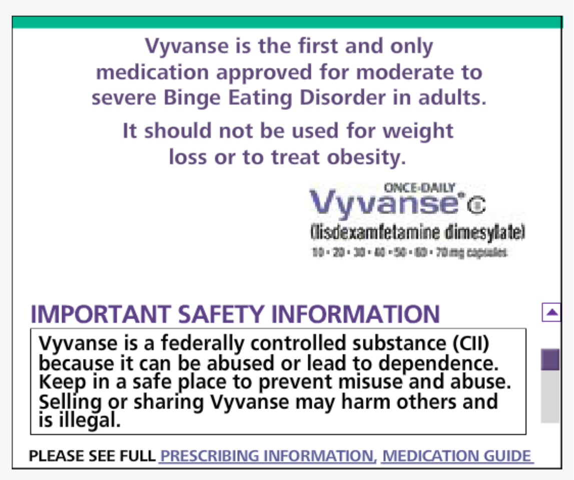

2015 version:

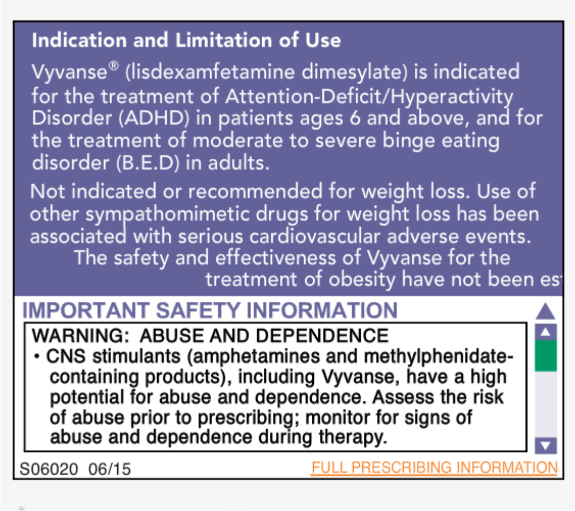

Alvesco

2011 version:

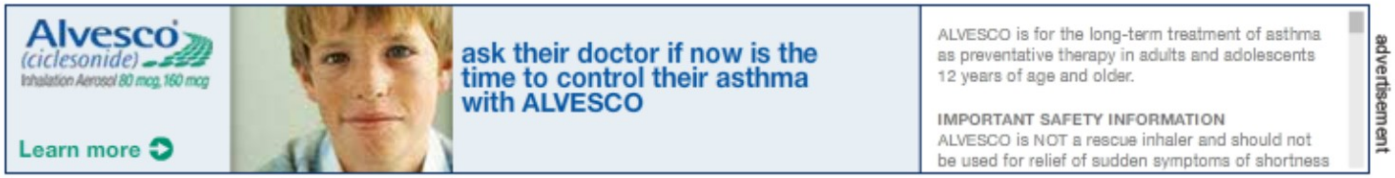

2014 version:

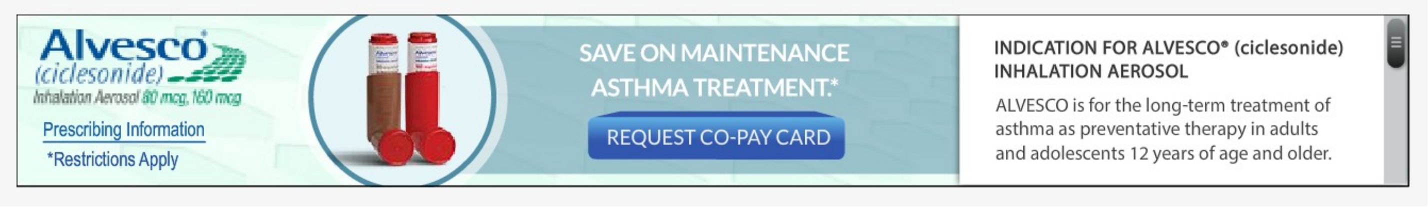

Amitiza

2011 version:

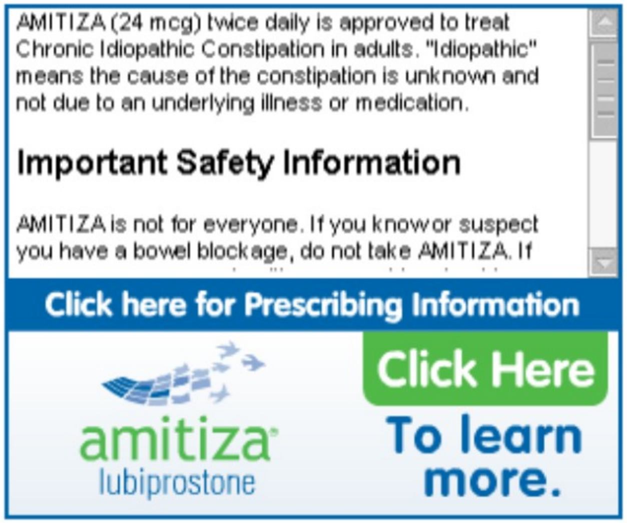

2013 version:

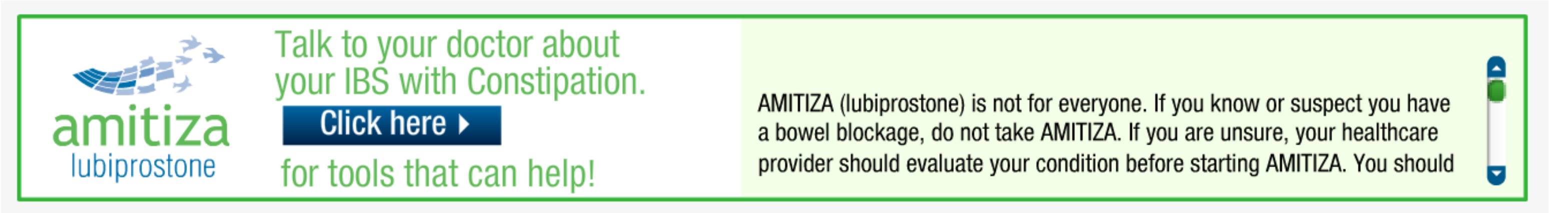

Cialis

2011 version:

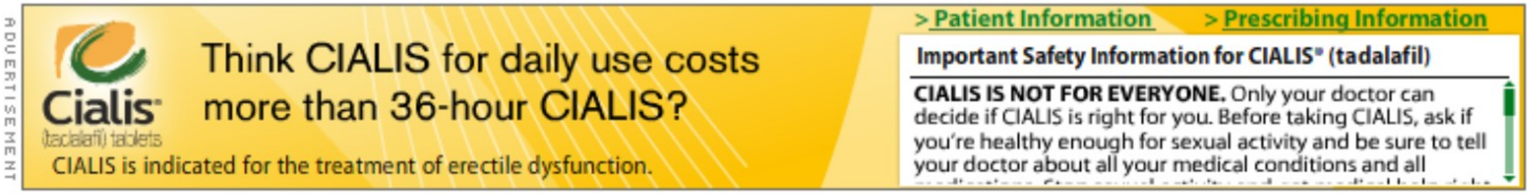

2015 version:

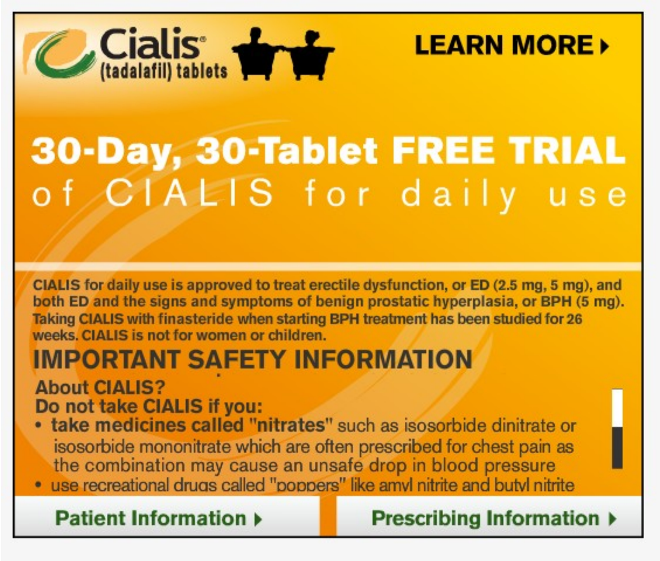

Cimzia

2011 version:

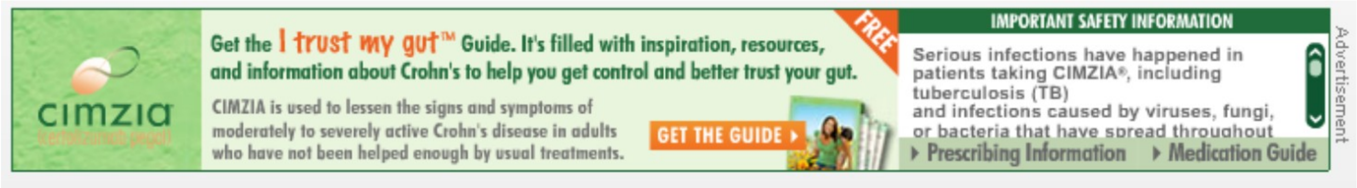

2013 version:

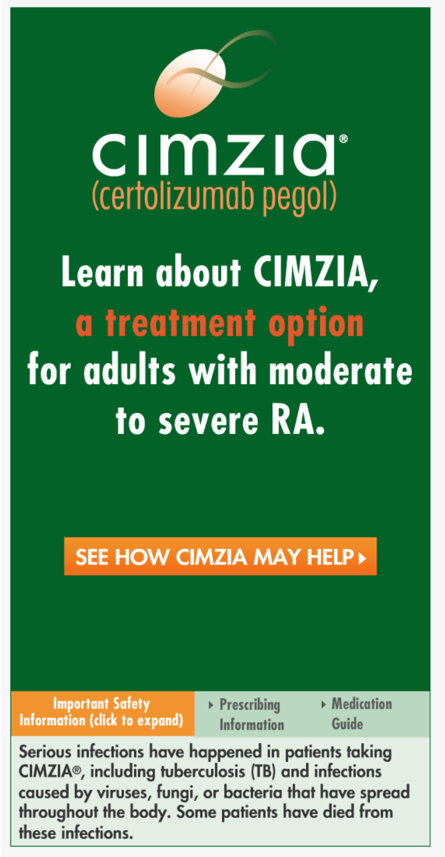

Supplement: Multimedia Appendix 3 [file jmir_v18i2e33_app3.pdf]
